# Disparate Clinical Characteristics and Prognosis of HFpEF versus HFrEF Phenotype of Diabetic Cardiomyopathy

**DOI:** 10.3390/jcm12041565

**Published:** 2023-02-16

**Authors:** Zheng Li, Yi Shi, Yiyuan Xia, Lida Wu, Hui Li, Rong Zhou, Xiaofei Gao, Hongsong Zhang, Xiaoping Jin, Junxia Zhang

**Affiliations:** 1 Department of Cardiology, Nanjing First Hospital, Nanjing Medical University, Nanjing 210006, China; 2 Department of Intensive Medicine, Qujing No. 1 Hospital, Yuanlin No. 1 Road, Qilin District, Qujing 655000, China

**Keywords:** diabetic cardiomyopathy, heart failure, phenotype

## Abstract

Aims: Diabetic cardiomyopathy (DCM) is an ill-defined entity. This study aims to explore the clinical characteristics and prognosis of diabetic patients that disparately develop heart failure (HF) with preserved ejection fraction (HFpEF) other than HF with reduced ejection fraction (HFrEF). Patients and Methods: A total of 911 patients diagnosed with diabetes mellitus were identified in the ChiHFpEF cohort (NCT05278026). DCM was defined as diabetic patients diagnosed with HF, absent from flow obstructive coronary artery disease (CAD), uncontrolled refractory hypertension and hemodynamics significant heart valvular diseases, arrhythmia and congenital heart diseases. The primary endpoint was a composite of all-cause death and rehospitalization due to HF. Results: As compared to DCM-HFrEF patients, DCM-HFpEF patients had a longer duration of diabetes, were older and more noticeable in hypertension and non-obstructive CAD. After a median follow-up of 45.5 months, survival analysis showed that DCM-HFpEF patients had a better composite endpoint. Cox regression implicated that non-obstructive CAD was a negative (HR 0.101, 95% CI 0.028–0.373, *p* = 0.001) predictor for the composite endpoint of DCM-HFrEF patients. Age was a positive predictor for the composite endpoint of DCM-HFpEF patients (HR 1.044, 95% CI 1.007–1.082, *p* = 0.018). Conclusion: DCM-HFpEF is a disparate entity from DCM-HFrEF. Additional phenomic studies are needed to explore the molecular mechanisms and develop targeted therapies.

## 1. Introduction

Diabetes mellitus (DM) is a metabolic disorder afflicting multiple systems and organs. It is considered as the seventh leading cause of death. Cardiovascular diseases (CVDs) are the main cause of deaths in DM patients. Even though advances in prevention and treatment of CVDs have greatly improved the prognosis of CVD patients, morbidity and mortality of CVD patients comorbid with DM remain high [1]. Additionally, matched for age, gender, hypertension and coronary artery disease (CAD), a cohort study discovered that participants with DM have an increased incidence of heart failure (HF) over a 10-year follow-up period [2]. Therefore, DM is recognized as a contributor for the development of HF independent of age, hypertension, obesity, hypercholesterolemia and CAD. Diabetic cardiomyopathy (DCM) was first described in 1972 as cardiomegaly and congestive HF without evidence of coronary artery or valve disease. The contemporary definition of DCM refers to cardiac dysfunction in diabetic patients in the absence of clinically significant coronary, valvular or hypertensive diseases [3].

Heart failure with preserved ejection fraction (HFpEF) and heart failure with reduced ejection fraction (HFrEF) are the two most common types of HF according to the updated classification of HF. However, clinical research of DCM-HFpEF versus DCM-HFrEF is lacking. It is deemed that in the early stage of DCM, left ventricular (LV) concentric remodeling, LV hypertrophy or diastolic dysfunction along with slightly decreased global longitudinal strain tend to be the primary abnormal manifestations [4]. These asymptomatic changes in cardiac structure and function gradually progress to systolic dysfunction, and eventually to clinical HF [5]. Therefore, our first question is whether DCM-HFpEF and DCM-HFrEF share most of the clinical features and DCM-HFpEF is a transitional state of non-DCM to DCM-HFrEF, or whether they are different clinical entities.

Studies comparing the clinical prognosis of HFrEF and HFpEF are controversial. Gregory et al. reported similar adjusted incidence rates of rehospitalization, but those with HFrEF had an increased risk of mortality at 30 days [6]. Others found that, among patients hospitalized with HF, HFpEF and HFrEF, patients had a comparably poor 5-year survival rate [7]. Comparative outcome studies of DCM-HFrEF versus DCM-HFpEF are scarce. In this study, we also analyzed the all-cause death and rehospitalization due to HF in non-DCM, DCM-HFrEF and DCM-HFpEF patients.

## 2. Methods

### 2.1. Study Design and Patient Selection

**Figure 1:**
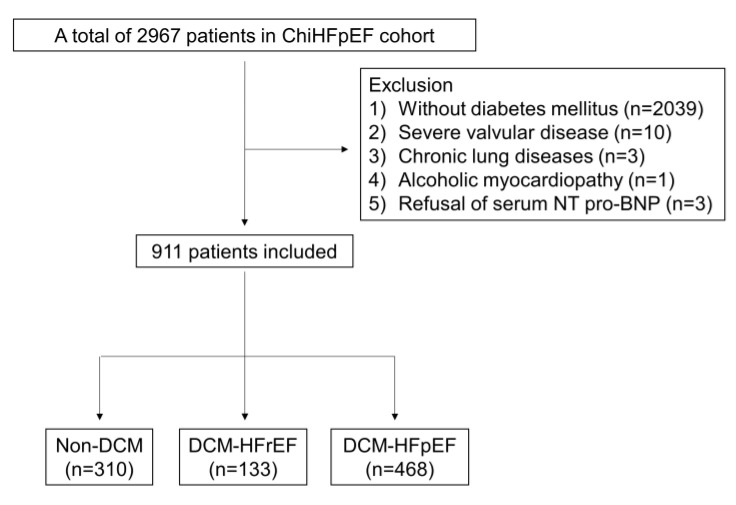
Patient flowchart. DCM: diabetic cardiomyopathy; HFrEF: heart failure with reduced ejection fraction; HFpEF: heart failure with preserved ejection fraction.

The ChiHFpEF cohort is a prospective study of HFpEF in 2967 Han Chinese patients with documented CVDs in Nanjing First Hospital (NCT05278026). After screening according to the inclusion and exclusion criteria, 911 patients from January 2014 to June 2022 with DM were included in the final analysis (Figure 1). Patients were recruited in this study if they were (1) ≥18 years old; (2) with type 2 diabetes (T2DM), which was confirmed if any of the following can be met: (a) fasting plasma glucose (FPG) ≥ 126 mg/dL (7.0 mmol/L), and fasting means no caloric intaking for at least 8 h; (b) 2 h plasma glucose (PG) ≥200 mg/dL (11.1 mmol/L) during oral glucose tolerance test (OGTT); (c) HbA1C ≥ 6.5% (48 mmol/mol); (d) suffering from hyperglycemic crisis or having classic symptoms of hyperglycemia, along with a random plasma glucose ≥200 mg/dL (11.1 mmol/L) [8]. Patients were excluded if they were (1) first diagnosed with type 1 diabetes (T1DM); diabetes secondary to other specific causes or women who were pregnant or breastfeeding; (2) patients with acute coronary syndromes or with hemodynamics significant (moderate to severe) heart valvular diseases; (3) patients with chronic lung diseases, aortic dissection, peripheral vascular diseases, pericardial diseases, myocarditis, hypertrophic cardiomyopathy, cardiophobia, costal chondritis, shock or thyroid diseases; (4) patients with infections in need of antibiotics; (5) patients with a previous history of malignancies.

HF was defined as the presence of at least one of the symptoms (dyspnea and fatigue) or signs (rales and ankle swelling) related to HF; NT pro-BNP ≥ 125 pg/mL with cardiac structural and/or functional dysfunction adjudicated by echocardiography. DCM was defined as diabetic patients with HF, who were free from flow obstructive CAD, uncontrolled refractory hypertension and hemodynamics significant heart valvular diseases, arrhythmia and congenital heart diseases. Taking HF with midrange ejection fraction (HFmrEF, LVEF 41–49%) as a mild form of HFrEF, we classified LVEF < 50% as HFrEF in the current analysis, while HFpEF was defined as LVEF ≥ 50% in accordance with 2021 European Society of Cardiology guidelines [9]. The echocardiographic inclusion criteria for HFpEF were as we previously described. (1) LAD > 40 mm; (2) E/E’ ≥ 13, E’/A’ < 1 [10]. All diabetic patients were divided into a non-DCM group, a DCM-HFrEF group and a DCM-HFpEF group in this study. Hypertension was confirmed as office blood pressure ≥ 140 mmHg systolic and/or 90 mmHg diastolic or demanding pharmacological treatment. Uncontrolled refractory hypertension was defined as blood pressure ≥ 160 mmHg systolic and/or 100 mmHg diastolic even after treatment with at least three pharmacological agents including diuretics. Non-obstructive CAD was defined as stenosis of main coronary arteries < 75% by percutaneous coronary angiography or coronary computed tomography (CT) angiography without indication of percutaneous coronary intervention. 

The study protocol and informed consent were approved by the institutional review committee of Nanjing First Hospital. Written informed consent for participation was obtained from all enrolled patients.

### 2.2. Outcome Measures

The primary endpoint of this study was a composite of all-cause death and rehospitalization due to HF during the follow-up period. The secondary endpoints were (1) all-cause death and (2) rehospitalization due to HF.

### 2.3. Clinical and Laboratory Variables

Patients’ fasting blood samples were collected rightly on the next day within 24 h after admission for further routine measurements of hematology, clinical chemistry, biomarkers of HF and myocardial injuries.

### 2.4. Cardiac Structure and Function Assessment by Echocardiography

The detailed collection and processing of echocardiographic data have been described before. In accordance with the internationally accepted guidelines, each patient included in the study underwent transthoracic echocardiography (TTE) with color flow Doppler and tissue Doppler by board-certified cardiologists trained in echocardiography. 

### 2.5. Statistical Analyses

All statistical analyses were performed with SPSS 25.0 (SPSS Inc., Chicago, IL, USA). In this study, DCM-HFpEF was considered as a reference group. Therefore, the primary analyses were performed in two approaches: (1) the non-DCM versus the DCM-HFpEF group and (2) the DCM-HFrEF versus the DCM-HFpEF group. Continuous values were expressed as mean ± standard deviation (SD) for data of normal distribution, or medians and the 25th to 75th interquartile range (IQR) for data of non-normal distribution. Categorical variables were expressed as numbers and percentages. Differences in numerical variables were analyzed using the Student’s *t*-test or Mann–Whitney U test. Categorical variables were analyzed by the Chi-square test or Fisher’s exact test. 

The cumulative hazard ratios of all-cause death, rehospitalization due to HF and composite endpoint in the DCM-HFpEF or DCM-HFrEF groups were estimated by Kaplan–Meier analysis and compared using the log-rank test. Cox proportional regression analyses were conducted to estimate the influence of risk factors on the outcomes of DCM-HFpEF or DCM-HFrEF. In the analysis of outcome predictors for HFrEF, we included age, gender, hypertension, non-obstructive CAD, duration of diabetes and heart rate (HR) at admission. In the analysis of the outcome predictors for HFpEF, age, hypertension, AF, pulmonary hypertension (PH), SBP and alcohol habit were included. The variables included in the Cox regression models for the primary endpoint (composite of all-cause death and rehospitalization due to HF) and secondary endpoints were chosen based on between-group univariate analysis. Statistical significance was a two-sided significance level of 0.05.

### 2.6. Trial Visits

The follow-up process was performed by outpatient visit or by telephone inquiry.

### 2.7. Patient and Public Involvement Statement

No patient was involved in the design of this study.

## 3. Results

### 3.1. Comparison of Baseline Characteristics between Non-DCM versus DCM-HFpEF Patients

**Figure 2:**
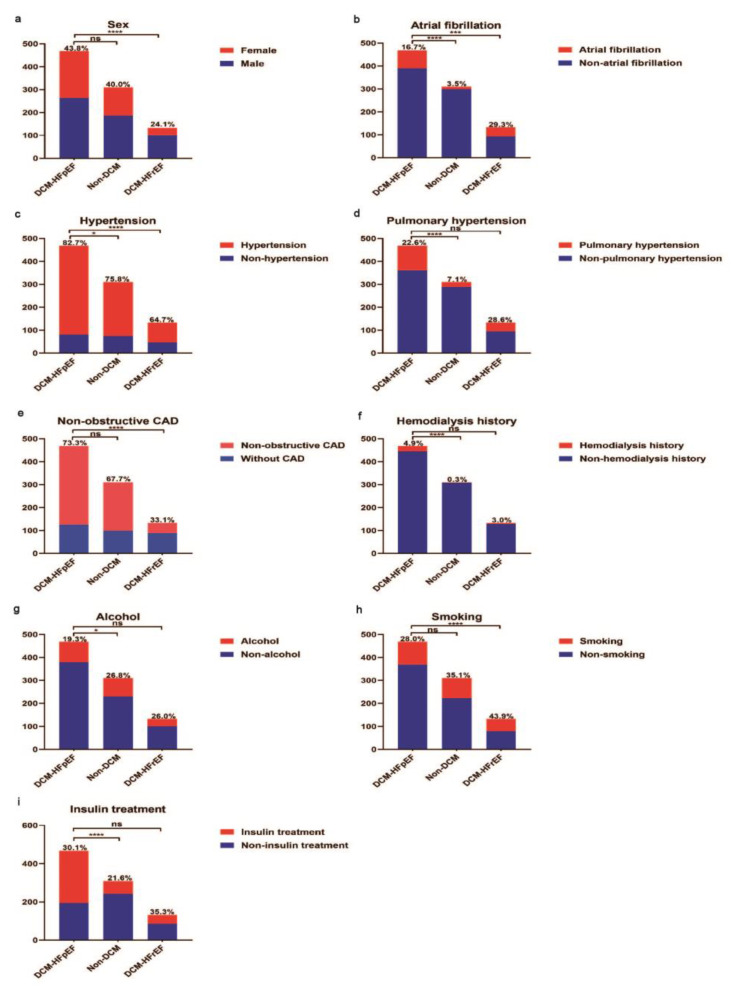
Comparison of demographic information during hospitalization of DCM-HFpEF vs. non-DCM and DCM-HFrEF vs. DCM-HFpEF patients. (**a**) Gender differences between DCM-HFpEF and non-DCM patients were not obvious. Compared with DCM-HFrEF patients, DCM-HFpEF patients were more likely to be female. (**b**) DCM-HFpEF patients had a higher proportion of atrial fibrillation vs. non-DCM and a lower proportion of atrial fibrillation vs. DCM-HFrEF. (**c**) Patients had a higher proportion of hypertension in DCM-HFpEF vs. non-DCM and DCM-HFrEF. (**d**) Patients had a higher proportion of pulmonary hypertension in DCM-HFpEF vs. non-DCM, but differences between DCM-HFpEF and DCM-HFrEF were not obvious. (**e**) Patients had a higher proportion of non-obstructive CAD in DCM-HFpEF vs. DCM-HFrEF. (**f**) Patients had a higher proportion of hemodialysis history in DCM-HFpEF vs. non-DCM. (**g**) DCM-HFpEF patients had a lower proportion of alcohol assumption vs. non-DCM. (**h**) DCM-HFrEF patients had a higher proportion of smoking assumption vs. DCM-HFpEF. (**i**) DCM-HFpEF patients had a higher proportion of insulin treatment vs. non-DCM. CAD: coronary artery disease; DCM: diabetic cardiomyopathy; HFrEF: heart failure with reduced ejection fraction; HFpEF: heart failure with preserved ejection fraction. * *p* < 0.05. *** *p* < 0.005. **** *p* < 0.001.

The baseline demographics, clinical presentation, and laboratory and echocardiographic results included in this study are presented in Table 1. Compared with non-DCM patients, DCM-HFpEF patients were older (65.5 ± 10.2 versus 70.2 ± 10.0, *p* < 0.001). The gender differences between the two groups were not obvious. However, DCM-HFpEF patients had a longer diabetes duration, a higher proportion of PH and were more likely to be treated with insulin and hemodialysis (Figure 2). There was no significant difference in HbA1c between the two groups. NT-proBNP, creatinine, urea and uric acid were significantly higher, whereas haemoglobin was significantly lower in DCM-HFpEF as compared to non-DCM patients. Patients with DCM-HFpEF displayed diastolic dysfunction as evidenced by a higher E/E’ ratio and larger left atrial diameter (LAD).

### 3.2. Comparison of Basic Characteristics between DCM-HFpEF and DCM-HFrEF Patients

Compared with DCM-HFrEF, DCM-HFpEF patients were older (70.2 ± 10.0 versus 65.1 ± 11.8, *p* < 0.001), and more likely to be female (43.8% versus 24.1%, *p* < 0.001). Interestingly, the disease course of diabetes was longer in DCM-HFpEF patients than that of DCM-HFrEF patients, 480 (192–696) weeks versus 240 (48–480) weeks, *p* < 0.001, indicating DCM-HFpEF is unlikely to be the transitional state between non-DCM to DCM-HFrEF. Furthermore, DCM-HFpEF patients had a higher proportion of hypertension (82.7% versus 64.7%, *p* < 0.001). They also had a slower HR and higher SBP at the time of admission. In addition, there was a higher proportion of non-obstructive CAD (73.3% versus 33.1%, *p* < 0.001) and a lower proportion of AF (16.7% versus 29.3%, *p* = 0.001) in DCM-HFpEF versus DCM-HFrEF patients. 

Patients with DCM-HFrEF had higher levels of NT-proBNP than patients with DCM-HFpEF, 2711 (915–9095) versus 558 (326–1152), *p* < 0.001. There were also elevated levels of alanine aminotransferase, aspartate aminotransferase, creatinine, urea, uric acid, LDL-C and haemoglobin in DCM-HFrEF patients compared with DCM-HFpEF patients. 

**Figure 3:**
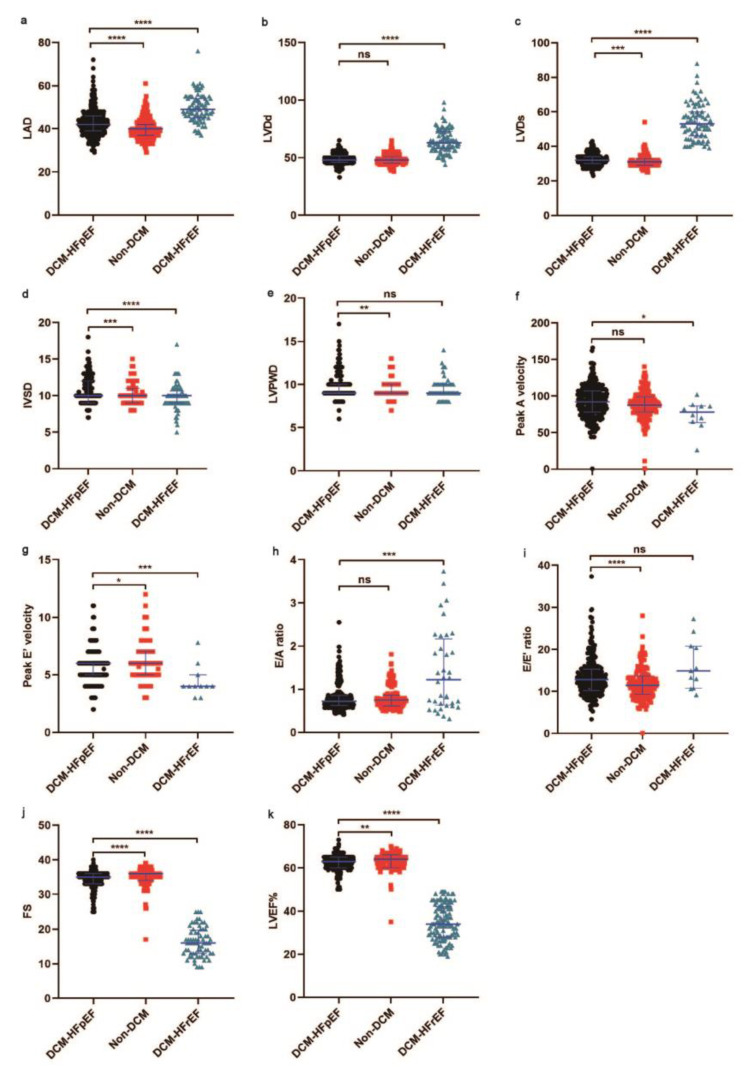
Comparison of echocardiography data during hospitalization of DCM-HFpEF vs. non-DCM and DCM-HFpEF vs. DCM-HFrEF. (**a**) LAD was higher in DCM-HFrEF vs. DCM-HFpEF and lower in non-DCM vs. DCM-HFpEF. (**b**,**c**) LVDd and LVDs were higher in DCM-HFrEF vs. DCM-HFpEF. (**d**) IVSD was higher in DCM-HFpEF vs. non-DCM and DCM-HFrEF. (**e**) LVPWD was higher in DCM-HFpEF vs. non-DCM. (**f**) Peak A velocity was higher in DCM-HFpEF vs. DCM-HFrEF. (**g**) Peak E’ velocity was higher in non-DCM vs. DCM-HFpEF and lower in DCM-HFrEF vs. DCM-HFpEF. (**h**) E/A ratio was higher in DCM-HFrEF vs. DCM-HFpEF. (**i**) E/E’ was lower in non-DCM vs. DCM-HFpEF. (**j**) FS was higher in non-DCM vs. DCM-HFpEF and lower in DCM-HFrEF vs. DCM-HFpEF. (**k**) LVEF% was higher in non-DCM vs. DCM-HFpEF and lower in DCM-HFrEF vs. DCM-HFpEF. LVEF: left ventricular ejection fraction; LAD: left atrial diameter; IVSD: ventricular septum diameter; LVPWD: left ventricular posterior wall diameter; LVDd: left ventricular diameter in diastole; LVDs: left ventricular diameter in systole; FS: fractional shortening; Peak A velocity: the maximum early transmitral flow velocity in atrial systole; Peak E velocity: the maximum early transmitral flow velocity; E/A ratio: the ratio of the early (E) to late (A) ventricular filling velocities; E/E’ ratio: the ratio of mitral peak velocity of the early filling (E) to early diastolic mitral annular velocity (E). DCM: diabetic cardiomyopathy; HFrEF: heart failure with reduced ejection fraction; HFpEF: heart failure with preserved ejection fraction. Black represents DCM-HFpEF, red represents non-DCM, green represents DCM-HFrEF. * *p* < 0.05. ** *p* < 0.01. *** *p* < 0.005. **** *p* < 0.001.

Compared with DCM-HFrEF, LV enlargement was not significant in DCM-HFpEF patients, showing as LVDd: 64 mm versus 48 mm, *p* < 0.001 and LVDs: 54 mm versus 32 mm, *p* < 0.001. Consistently, LA enlargement was more salient in DCM-HFpEF versus DCM-HFrEF, 49 mm versus 42 mm, *p* < 0.001. This was in accordance with a higher incidence of atrial fibrillation in DCM-HFrEF patients. Systolic function was largely preserved in DCM-HFpEF patients in comparison to DCM-HFrEF patients, 33% versus 64%, *p* < 0.001. The main differences in echocardiographic parameters are visualized in Figure 3.

### 3.3. Survival Analysis of DCM-HFrEF, DCM-HFpEF and Non-DCM Patients

**Figure 4:**
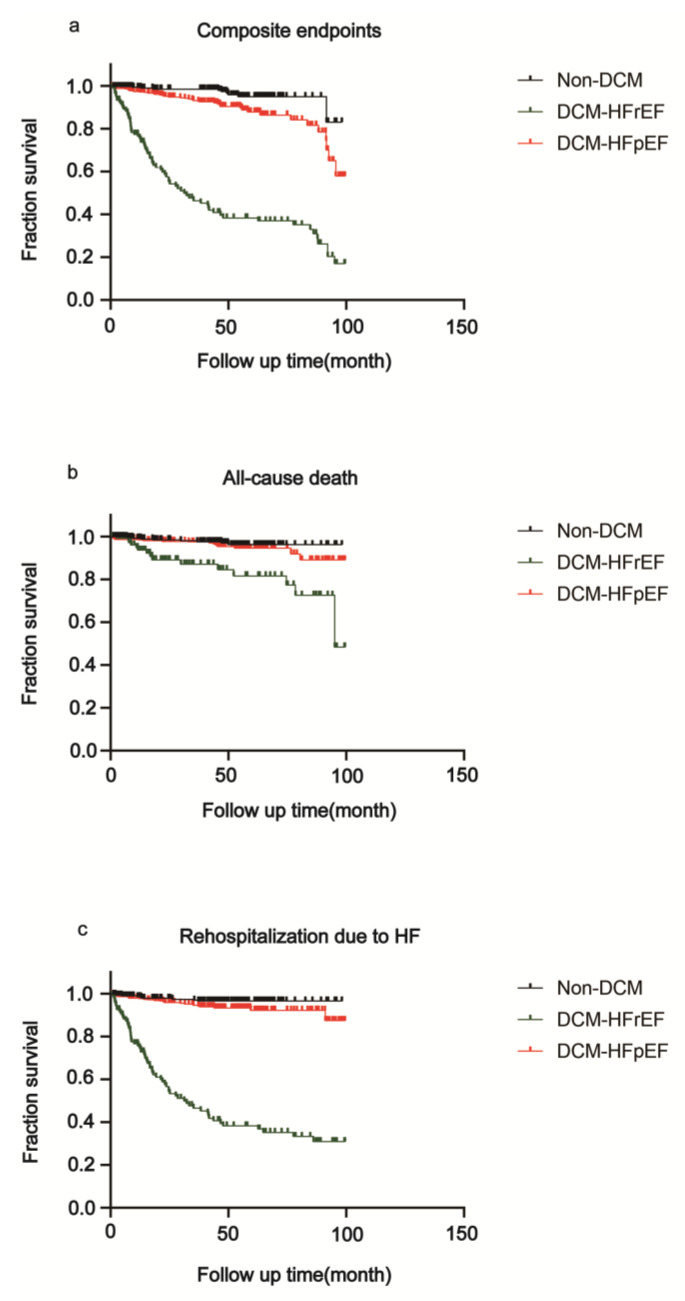
Kaplan–Meier survival analysis after a median follow-up of 45.4 months. Kaplan–Meier estimates for a composite of all-cause death and rehospitalization due to HF among DCM-HFpEF, non-DCM and DCM-HFrEF patients. (**a**) HFrEF showed the lowest event-free rate for composite endpoints, followed by DCM-HFpEF. (**b**) HFrEF showed the lowest event-free rate for all-cause death, followed by DCM-HFpEF. (**c**) HFrEF showed the lowest event-free rate for rehospitalization due to HF, followed by DCM-HFpEF. DCM: diabetic cardiomyopathy; HFrEF: heart failure with reduced ejection fraction; HFpEF: heart failure with preserved ejection fraction.

After a median follow-up of 45.5 months (interquartile range 23.2–62.3), 122 patients (13.4%) reached the clinical endpoints presented in Figure 4, Table 2. All-cause death occurred in 41 patients, and 100 patients were readmitted due to HF. Deaths occurred in 17 (3.6%) DCM-HFpEF patients versus 8 (2.6%) non-DCM patients (HR 0.60, 95% CI 0.27–1.33, *p* = 0.2074). A total of 25 (5.3%) rehospitalizations due to HF occurred in DCM-HFpEF patients and 9 (2.9%) occurred in non-DCM groups (HR 0.52, 95% CI 0.26–1.03, *p* = 0.0615). The composite endpoints occurred in 41 (8.8%) DCM-HFpEF patients versus 10 (3.2%) non-DCM patients (HR 0.38, 95% CI 0.22–0.66, *p* = 0.0021). 

In patients of DCM-HFrEF, 16 (12.0%) deaths occurred (HR 2.99, 95% CI 1.37–6.55, *p* = 0.0061), compared with DCM-HFpEF patients. A total of 66 (49.6%) rehospitalizations due to HF occurred in DCM-HFrEF patients (HR 30.46, 95% CI 18.07–51.32, *p* < 0.0001), compared with DCM-HFpEF patients. The composite endpoints occurred in 71 (53.4%) DCM-HFrEF patients (HR 11.76, 95% CI 7.47–18.52, *p* = 0.0021) as compared to DCM-HFpEF patients. 

**Figure 5:**
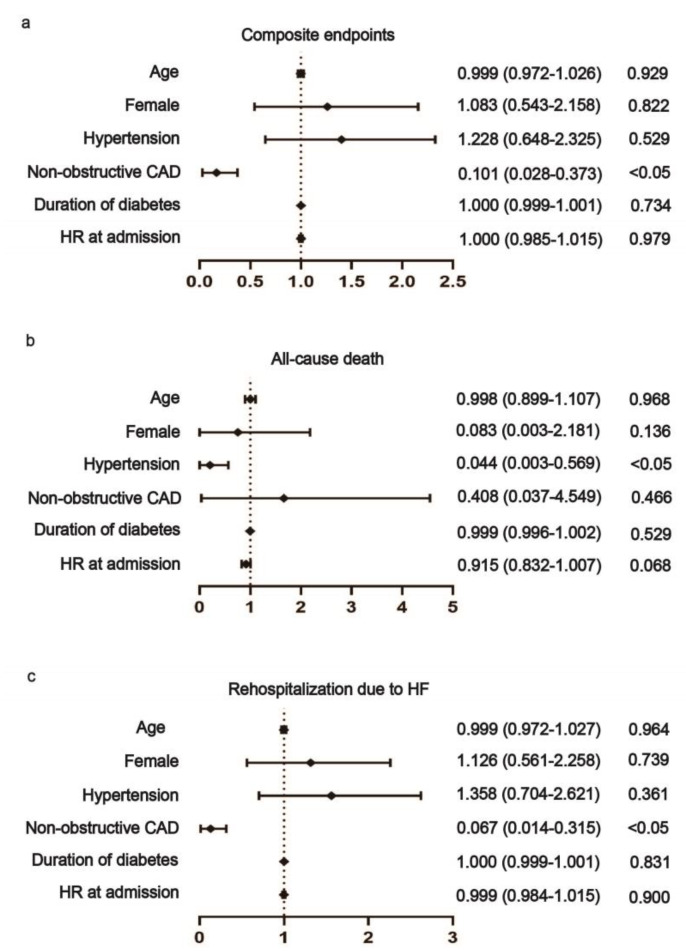
Association between risk factors and prognosis in DCM-HFrEF patients. Non-obstructive CAD was a negative predictor for both composite endpoint (**a**) and rehospitalization due to HF (**c**). Hypertension was a negative predictor for death of DCM-HFrEF patients (**b**). CAD: coronary artery disease; HR: heart rate.

**Figure 6:**
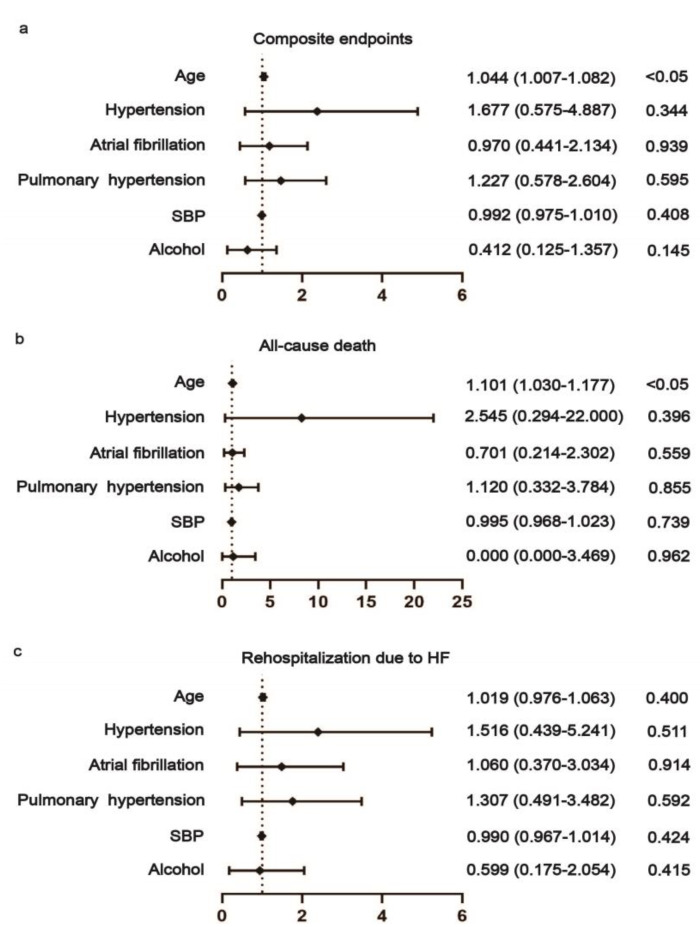
Association between risk factors and prognosis in DCM-HFpEF patients. Age was a positive predictor for both a composite endpoint (**a**) and death (**b**) of DCM-HFpEF patients. SBP: systolic blood pressure. (**c**) No association was found between these risk factors and prognosis in DCM-HFpEF patients.

Multivariable Cox proportional hazards regression implicated that non-obstructive CAD was a negative predictor for both composite endpoint (HR 0.101, 95% CI 0.028–0.373, *p* = 0.001) and rehospitalization due to HF (HR 0.067, 95% CI 0.014–0.315, *p* = 0.001) of DCM-HFrEF patients (Figure 5). Hypertension was also a negative predictor for the death of DCM-HFrEF patients (HR 0.044, 95% CI 0.003–0.569, *p* = 0.017) (Figure 5). Age was a positive predictor for both composite endpoint (HR 1.044, 95% CI 1.007–1.082, *p* = 0.018) and death (HR 1.101, 95% CI 1.030–1.177, *p* = 0.004) of DCM-HFpEF patients (Figure 6).

## 4. Discussion

In this study, we conducted a prospective, single-center cohort study of DCM according to the different phenotypes of HFrEF versus HFpEF, in order to explore the potential clinical features related to the heterogeneity of DCM. The main findings are (1) compared with DCM-HFrEF patients, DCM-HFpEF patients were older and more likely to be female, suggesting the disparate demographic characteristics of DCM-HFrEF versus DCM-HFpEF patients; (2) DCM-HFpEF patients had longer disease courses of diabetes versus non-DCM and DCM-HFrEF patients, indicating DCM-HFpEF is not a transitional state between non-DCM and DCM-HFrEF; (3) All-cause death and rehospitalization due to HF were more frequent in DCM-HFrEF versus DCM-HFpEF patients; (4) age was a positive predictor for a composite endpoint of DCM-HFpEF versus non-DCM patients; (5) non-obstructive CAD was a negative predictor for both a composite endpoint and rehospitalization due to HF of DCM-HFrEF patients. Hypertension was also a negative predictor for death of DCM-HFrEF patients; (6) age was a positive predictor for both a composite endpoint and death.

The classification of HF according to left ventricular ejection fraction (LVEF) was recommended recently [9]. HFpEF is characterized as an LVEF ≥ 50%, with symptoms and signs of HF and LV diastolic dysfunction. The HFpEF patients tend to have high prevalence of metabolic disorders and diabetes mellitus and are more noticeable in the aging population [11,12]. Multiple factors, such as systemic inflammatory responses, metabolic disorders, epicardial adipose tissue (EAT) accumulation and myocardial fibrosis have been shown to contribute to the development of HFpEF [13]. Heart failure with reduced and mildly reduced ejection fraction (HFrEF, EF < 40% and HFmrEF, EF 40–49%) were combined in this study. HFpEF is distinct from HFrEF in some respects. Taking history and physical examination into consideration, research based on PARAGON-HF (Prospective Comparison of ARNI with ARB Global Outcomes in HF with Preserved Ejection Fraction) uncovered that edema was common in patients with HFpEF while a third heart sound was frequent in those with HFrEF, providing irreplaceable prognostic information [14]. However, in DCM patients, clinical characteristics of different HF phenotypes remain unexplored. 

DCM is primarily caused by detrimental mechanisms in the myocardium by elevated serum glucose, hyperinsulinemia and glycosylated proteins, leading to capillary damage, myocardial hypertrophy with mitochondrial dysfunction and myocardial fibrosis [15,16]. It is obviously different from myocardial ischemia/infarction or abnormal hemodynamic loads. Therefore, DM patients comorbid with flow obstructive CAD, refractory hypertension and heart diseases with an abnormal hemodynamic state were excluded according to the definition of DCM in this study. The level of glycosylated hemoglobin, type A1C (HbA1c) and fasting glucose were similar in three groups. However, the prevalence of aging, female gender, hypertension and non-obstructive CAD were higher in DCM-HFpEF versus DCM-HFrEF patients. The duration of diabetes was longer and systolic blood pressure at admission was higher in DCM-HFpEF versus DCM-HFrEF patients. It is worth mentioning that the longer diabetes duration of DCM-HFpEF patients implies that DCM-HFpEF is not the transitional state between non-DCM and DCM-HFrEF patients. Diabetic patients might progress in different directions as DCM-HFpEF or DCM-HFrEF patients. 

In our study, the prevalence of AF, PH, use of insulin and hemodialysis history were higher in DCM-HFpEF and DCM-HFrEF patients as compared to non-DCM patients. Renal dysfunction has been confirmed to accelerate the progression of HF and to increase the risk of hospitalization, rehospitalization, intensive condition and death [17]. In this condition, diabetic kidney disease, which is a microvascular complication, gradually worsened and ultimately hemodialysis or renal transplantation was needed [18]. It was in accordance with our findings that both DCM-HFrEF and DCM-HFpEF participants had a higher percentage of hemodialysis treatment. 

Moreover, significant negative cardiac remodeling was noticed in the DCM-HFrEF group as compared to the DCM-HFpEF group. We previously reported NT-proBNP was not associated with diastolic dysfunction in HFpEF patients [19]. However, there is a lack of sensitive predictors of diastolic dysfunction for HFpEF patients. Lower mortality in HFpEF patients compared with HFrEF patients was reported [20,21]. Smith et al. [22] showed that HFrEF patients had higher mortality than patients with HFpEF during six months of follow-up. Survival analysis of our data indicated that there was an obvious difference as to all-cause death, rehospitalization due to HF and composite endpoints in three groups. DCM-HFrEF patients had the poorest prognosis. Overall, the event rates in DCM-HFpEF patients are much lower than expected. Solomon et al. found that over a median of 2.3 years, a composite of worsening heart failure and cardiovascular death occurred in 19.5% of the placebo group [23]. Likewise, the EMPEROR-Preserved trial indicated that over a median of 26.2 months, a composite of cardiovascular death or hospitalization for heart failure occurred in 17.1% of the placebo group [24]. All these event rates in HFpEF patients were higher compared to our study. According to the definition of diabetic cardiomyopathy, we excluded diabetic patients with coronary heart diseases with severe lesions, valvular heart diseases, etc. The event rate of HFpEF patients with severe coronary heart diseases might be higher than DCM-HFpEF patients. Therefore, it is possible that event rates of DCM-HFpEF patients are lower than HFpEF patients due to other etiologies.

It remains unknown whether the comorbidities and traditional risk factors such as hypertension, CAD and aging are merely concurrent or contribute to the disparate development of DCM-HFpEF versus DCM-HFrEF phenotypes. Mechanistically, metabolic inflammation underlying hyperglycemia and insulin resistance could lead to a direct detrimental effect on cardiomyocytes and cause impaired calcium homeostasis, oxidative stress, mitochondrial dysfunction, apoptosis, myocardial fibrosis and myocardial remodeling [25,26,27]. Recently, we demonstrated that pyroptosis of adipocytes from epicardial adipose tissue participated in the myocardial insults in the hypertensive and high-fat diet-fed animal model of HFpEF [28]. However, metabolic inflammation is a shared mechanism that might drive DCM patients to develop both HFpEF and HFrEF. Thus far, knowledge about the mechanisms dictating dichotomous DCM phenotypes of HFpEF versus HFrEF is lacking. 

It is of interest that there is a much lower incidence of hypertension and non-obstructive CAD in DCM-HFrEF versus DCM-HFpEF patients in this study. Furthermore, non-obstructive CAD was a protective factor as to composite endpoints for DCM-HFrEF, largely owing to reducing rehospitalization due to HF. Although it needs to be confirmed in a large clinical cohort of DCM, we showed for the first time the lower incidence of hypertension and non-obstructive CAD in DCM-HFrEF versus DCM-HFpEF patients. Our observation of different incidences of CAD and hypertension might shed insight into the mechanisms of differential development of DCM-HFrEF versus DCM-HFpEF. 

HFpEF was mainly caused by aging, endothelial and microcirculatory dysfunction, as well as low-grade inflammation but not a severe hit of myocardial ischemia. In our study, we depicted the clinical facts such as non-obstructive CAD and hypertension that correlate to DCM-HFpEF and DCM-HFrEF. We did not explore the genetic susceptibilities of DCM-HFpEF and DCM-HFrEF which may play an important part in explaining why non-obstructive CAD and hypertension have a major role in HFrEF but not in HFpEF.

In a study of HFpEF phenogroups, the phenogroup with a high incident of DM had the comorbidities of obesity and a high level of renin. Renin and the prorenin receptor participate in blood pressure regulation, adipogenesis, glucose and insulin resistance and lipid homeostasis [29,30,31]. Patients with hypertension have relatively higher levels of plasma renin and plasma renin activity (PRA) [32]. Furthermore, PRA had the potential to guide renal denervation in hypertension patients [33]. A study found that despite similar baseline blood pressure, treatment response of renal denervation was significantly greater for patients with baseline PRA ≥0.65 ng/mL/h versus <0.65 ng/mL/h [34], indicating PRA might be a surrogate marker of nerval system regulating hypertension. Our group previously reported surgical renal denervation improved the cardiac function of experimental DCM [35]. The renin-angiotensin-aldosterone system (RAAS) is activated in DM [26]. This is evident both systemically and within the heart and across both clinical and preclinical contexts. As the RAAS plays a critical role in the regulation of blood pressure [36,37], both the resultant increased afterload secondary to DM–induced systemic RAAS upregulation and the direct actions of the cardiac RAAS on the myocardium contribute to DM–induced remodeling. Renin is supposed to mediate the reciprocal mechanisms consisting of the heart, kidney and nerval system and might participate in the development of DCM-HFpEF. The role of renin in the pathogenesis of DCM-HFpEF versus DCM-HFrEF needs to be investigated in future clinical and experimental studies. 

## 5. Limitations

There are some limitations of the study. To begin with, the study findings should be interpreted with caution due to the small sample size and an unclear risk of selection bias. Next, the samples were not evenly grouped. Meanwhile, residual and uncontrolled confounding were unavoidable in this study. Therefore, the conclusion drawn in this study needs to be confirmed by multi-center, large-scale studies.

## 6. Conclusions

Factors related to macrovascular atherosclerosis, hypertension and gender might drive the development of DCM-HFrEF; age might be a predictor of DCM-HFpEF. As to their clinical outcome, DCM-HFrEF patients had the highest mortality, followed by DCM-HFpEF patients.

## Figures and Tables

**Table 1:** Baseline characteristics of non-DCM, DCM-HFrEF and DCM-HFpEF.

Variables	DCM-HFpEF (n = 468)	Non-HF (n = 310)	DCM-HFrEF (n = 133)	*p* Value *	*p* Value †
Age, y	70.2 ± 10.0	65.5 ± 10.2	65.1 ± 11.8	<0.001	<0.001
Female, n(%)	205 (43.8)	124 (40.0)	32 (24.1)	0.293	<0.001
Hypertension, n(%)	387 (82.7)	235 (75.8)	86 (64.7)	0.019	<0.001
Non-obstructive coronary heart disease, n(%)	343 (73.3)	210 (67.7)	44 (33.1)	0.095	<0.001
Atrial fibrillation, n(%)	78 (16.7)	11 (3.5)	39 (29.3)	<0.001	0.001
Pulmonary hypertension, n(%)	106 (22.6)	22 (7.1)	38 (28.6)	<0.001	0.158
Use of insulin, n(%)	274 (30.1)	67 (21.6)	47 (35.3)	<0.001	0.805
Duration of diabetes, week	480 (192–696)	240 (96–480)	240 (48–480)	<0.001	<0.001
Hemodialysis history, n(%)	23 (4.9)	1 (0.3)	4 (3.0)	<0.001	0.349
Alcohol habit, n(%)	88 (19.3)	80 (26.8)	32 (26.0)	0.016	0.105
Smoking habit, n(%)	99 (28.0)	87 (35.1)	54 (43.9)	0.063	0.001
HR at admission, bpm	72 (67–79)	72 (67–78)	82 (72–95)	0.920	<0.001
SBP at admission, mmHg	130 (124–145)	130 (120–140)	129 (120–140)	0.182	<0.001
DBP at admission, mmHg	79 (70–80)	80 (70–80)	80 (70–84)	0.397	0.301
NT-proBNP, pg/mL	558 (326–1152)	28 (18–42)	2711 (915–9095)	<0.001	<0.001
HbA1c, %	7.1 (6.4–7.9)	7.0 (6.2–8.1)	7.1 (6.3–8.2)	0.896	0.568
Alanine aminotransferase, U/L	18 (13–26)	20 (15–31)	23 (14–36)	<0.001	<0.001
Aspartate aminotransferase, U/L	19 (16–24)	19 (16–24)	22 (18–30)	0.186	<0.001
Potassium, mmol/L	3.9 (3.6–4.1)	3.9 (3.6–4.1)	4.0 (3.6–4.2)	0.896	0.119
Sodium, mmol/L	141 (139–142)	141 (139–143)	140 (138–142)	0.026	0.135
Chlorine, mmol/L	103 (101–106)	104 (101–106)	103 (100–105)	0.402	0.080
Creatinine, umol/L	78 (64–108)	67 (56–81)	91 (75–125)	<0.001	<0.001
Urea, mmol/L	6.3 (5.1–8.8)	5.7 (4.6–6.9)	8.0 (6.4–11.1)	<0.001	<0.001
Uric acid, umol/L	338 (267–434)	309 (250–365)	432 (346–579)	0.003	<0.001
Glucose, mmmol/L	6.8 (5.5–8.7)	7.0 (6.0–8.4)	7.4 (5.8–9.1)	0.239	0.095
Total cholesterol, mmol/L	3.64 (3.02–4.47)	3.82 (3.13–4.57)	3.65 (2.93–4.38)	0.103	0.883
Triglycerides, mmol/L	1.39 (1.00–1.97)	1.54 (1.06–2.22)	1.15 (0.92–1.57)	0.010	<0.001
HDL-C, mmol/L	0.97 (0.84–1.13)	0.97 (0.83–1.14)	0.92 (0.75–1.08)	0.809	0.115
LDL-C, mmol/L	2.03 (1.53–2.64)	2.11 (1.61–2.70)	2.23 (1.72–2.82)	0.322	0.010
Lipoprotein A1, mg/L	1.20 (1.07–1.35)	1.22 (1.05–1.35)	1.12 (0.89–1.27)	0.573	<0.001
Lipoprotein B, mg/L	0.71 (0.56–0.90)	0.76 (0.60–0.93)	0.74 (0.59–0.87)	0.078	0.540
Lpa, mg/L	166 (86–388)	129 (66–315)	162 (91–308)	0.016	0.762
Haemoglobin, g/dl	127 (115–138)	135 (124–143)	135 (120–147)	<0.001	<0.001
White blood cells, 109/L	6.48 (5.24–7.67)	6.49 (5.61–7.69)	6.79 (5.57–8.15)	0.434	0.064
LVEF, %	64 (62–65)	65 (63–66)	33 (28–39)	0.008	<0.001
AOD, mm	33 (31–36)	33 (31–36)	33 (31–36)	0.650	0.097
LAD, mm	42 (39–46)	40 (31–42)	49 (45–54)	<0.001	<0.001
LVDd, mm	48 (46–51)	48 (45–51)	64 (58–73)	0.081	<0.001
LVDs, mm	32 (30–34)	31 (29–33)	54 (47–63)	0.001	<0.001
IVSD, mm	10.0 (9.0–12.0)	10.0 (9.0–11.0)	9.5 (9.0–10.0)	0.001	<0.001
LVPWD, mm	9.0 (9.0–10.0)	9.0 (9.0–10.0)	9.0 (9.0–10.0)	0.006	0.089
FS, %	35 (33–36)	36 (34–36)	16 (13–18)	<0.001	<0.001
SV, ml	70 (60–77)	70 (60–77)	64 (41–71)	0.363	0.549
Peak E velocity, cm/s	70 (57–85)	65 (56–77)	58 (51–87)	0.101	0.522
Peak A velocity, cm/s	92 (78–107)	88 (79–99)	78 (64–87)	0.116	0.018
Peak E’ velocity, cm/s	6.0 (5.0–6.0)	6.0 (5.0–7.0)	4.0 (3.8–4.3)	0.011	0.004
Peak A’ velocity, cm/s	10.0 (8.0–11.0)	10.0 (9.0–11.0)	9.5 (7.4–10.8)	0.402	0.637
E/A ratio	0.72 (0.63–0.86)	0.74 (0.61–0.86)	0.79 (0.59–1.30)	0.566	0.002
E’/A’ ratio	0.60 (0.50–0.70)	0.62 (0.50–0.71)	0.67 (0.55–0.80)	0.244	0.064
E/E’ ratio	12.80 (10.20–15.31)	11.43 (9.29–13.67)	14.38 (10.71–21.63)	<0.001	0.108

Normally distributed data presented as mean (SD), and non-normally distributed data presented as median (interquartile range). * *p* is the value for the comparison between DCM-HFpEF and Non-HF, † *p* is the value for the comparison between DCM-HFpEF and DCM-HFrEF. HR: heart rate; SBP: systolic blood pressure; DBP: diastolic blood pressure; NT-proBNP: N-Terminal pro B-type natriuretic peptide; ST2: growth stimulation gene-2; HbA1c: glycosylated hemoglobin, type A1C; LDL-C: low-density lipoprotein-cholesterol; HDL-C: high-density lipoprotein cholesterol; LVEF: left ventricular ejection fraction; AOD: aortic diameter; LAD: left atrial diameter; IVSD: ventricular septum diameter; LVPWD: left ventricular posterior wall diameter; LVDd: left ventricular diameter in diastole; LVDs: left ventricular diameter in systole; FS: fractional shortening; SV: stroke volume; Peak A: the maximum early transmitral flow velocity in atrial systole; Peak A’: the maximum myocardial tissue velocity measured at the mitral annulus in atrial systole; Peak E: the maximum early transmitral flow velocity; Peak E’: myocardial tissue velocity measured at the septal and/or lateral mitral annulus.

**Table 2:** Clinical outcomes at 45.5 months.

Clinical Outcomes	Group	Number (%)	HR (95% CI)	*p* Value
Primary end point				
Composite endpoints	DCM-HFpEF (n = 468)	41 (8.8)	Reference	…
	Non-DCM (n = 310)	10 (3.2)	0.38 (0.22–0.66)	0.0021
	DCM-HFrEF (n = 133)	71 (53.4)	11.76 (7.47–18.52)	<0.0001
Secondary end point				
All-cause death	DCM-HFpEF (n = 468)	17 (3.6)	Reference	…
	Non-DCM (n = 310)	8 (2.6)	0.60 (0.27–1.33)	0.2074
	DCM-HFrEF (n = 133)	16 (12.0)	2.99 (1.37–6.55)	0.0061
Rehospitalization due to HF	DCM-HFpEF (n = 468)	25 (5.3)	Reference	…
	Non-DCM (n = 310)	9 (2.9)	0.52 (0.26–1.03)	0.0615
	DCM-HFrEF (n = 133)	66 (49.6)	30.46 (18.07–51.32)	<0.0001

## Data Availability

Data were available under the permission of corresponding authors.

## Abbreviations

DCM: diabetic cardiomyopathy; HFpEF: heart failure with preserved ejection fraction; HFrEF: heart failure with reduced ejection fraction.
